# Effects of workplace measures against COVID-19 on psychological distress of full-time employees: A 12-month prospective study in the COVID-19 pandemic

**DOI:** 10.5271/sjweh.4030

**Published:** 2022-08-31

**Authors:** Hiroki Asaoka, Natsu Sasaki, Kotaro Imamura, Reiko Kuroda, Kanami Tsuno, Norito Kawakami

**Affiliations:** 1 Department of Psychiatric Nursing, Graduate School of Medicine, The University of Tokyo, Tokyo, Japan; 2 Department of Mental Health, Graduate School of Medicine, The University of Tokyo, Tokyo, Japan; 3 Department of Public Mental Health Research, National Institute of Mental Health, National Center of Neurology and Psychiatry, Tokyo, Japan; 4 Division for Environment, Health and Safety, The University of Tokyo, Tokyo, Japan; 5 School of Health Innovation, Kanagawa University of Human Services, Kanagawa, Japan; 6 Junpukai Foundation, Okayama, Japan

**Keywords:** depression, employee, infection control measure, mental health, novel coronavirus disease, workplace

## Abstract

**Objective:**

This study aimed to investigate the prospective effects of corporate and organizational workplace measures against COVID-19 on reducing employees’ psychological distress during a 12-month follow-up in the COVID-19 pandemic.

**Methods:**

Data were retrieved from an online longitudinal panel survey of full-time employees in Japan, with the 1^st^ survey in March 2020, and the 2^nd^ to 6^th^ surveys in May, August, November 2020, February and March 2021, respectively. Seven area-specific workplace measures were assessed using a self-report 23-item scale at the 2^nd^ follow-up. Psychological distress was measured using an 18-item scale of the Brief Job Stress Questionnaire at each survey. Linear regressions and mixed model analysis were conducted of psychological distress at follow-ups on scores of the area-specific workplace measures, adjusting for psychological distress and other covariates at the 1^st^ survey.

**Results:**

A total of 941 employees responded at baseline; most of them (86.9–90.9%) participated in the follow-up surveys. Linear regression analysis indicated that workplace measures of facilitating employees’ preventive measures (ie, hygiene behaviors) statistically significantly and negatively correlated with psychological distress at the 5^th^ survey [b=-0.518, standard error (SE) 0.259, P=0.046]. A statistically significant and negative interaction between the scores and time of follow-up was observed in the mixed model analysis (b=-0.096, SE 0.047, P=0.041). No such correlation or interaction was found for any of other subcategorical workplace measures.

**Conclusions:**

The study provides prospective evidence for a protective effect of workplace measures to facilitate employee’s hygiene behaviors on reducing psychological distress of full-time employees in the COVID-19 pandemic. The association seems stronger at a later follow-up.

Poor mental health status has been reported to be a major public health problem in the global pandemic of the novel coronavirus disease (COVID-19) (1–3). Higher prevalence of psychiatric symptoms, such as depression, anxiety, post-traumatic stress symptoms, and insomnia, have been reported not only among community residents but also in the working populations including both healthcare workers and non-healthcare workers (4–6).

Among organizational and work-related interventions that have been proposed to mitigate the impact of the COVID-19 pandemic on the mental health of employees (7, 8), the adoption of correct preventive measures against COVID-19, including regular personal protective equipment supply and improved workplace infrastructure, is proposed as a potentially effective approach. The measures against COVID-19 in the workplace, such as disinfection of the work environment, encouraging telework and telecommuting and, encouraging mask wearing were reported as important preventive measures of COVID-19 (9, 10). During the 2009 influenza A (H1N1) pandemic, a randomized controlled trial reported that the adoption of combined workplace measures, including the measurements of body temperature each day and the obligation for symptomatic workers to stay at home, reduced the overall risk for infection transmission by 20% in the workplace (11). A previous study of employees who returned to work after the COVID-19 pandemic reported that personal preventive practices, such as hand hygiene and wearing face masks, as well as organizational measures including improvement of workplace hygiene from the company, were associated with less severe psychiatric symptoms (12). We also reported in a cross-sectional study of full-time employees in Japan that the number of workplace measures against COVID-19 infection implemented in companies/organizations was negatively associated with psychological distress (13). Another previous cross-sectional study reported a similar finding (14). However, these studies were inadequate to identify a temporal association because of their cross-sectional study design. It is unclear whether the effect of the workplace measures against COVID-19 on the improved mental health of employees was just short-term, limited to an early phase of the pandemic, or could remain for a longer period over the course of the pandemic. It is also unclear what types of areas of the workplace measures could be most effective in improving the mental health of employees, for instance, encouraging preventive measures taken by individuals, measures taken to reduce the risk of infection in the workplace, or information dissemination. Our previous study failed to show statistically significant associations between types measures and psychological distress (13). The items were arranged into seven categories in the previous study: (i) preventive measures taken by individuals; (ii) preventive measures taken to reduce the risk of infection in the workplace; (iii) criteria and procedure for waiting at home and clinical contact; (iv) temporary leave when infected or pandemic; (v) information about accommodation of high-risk people; (vi) introduction of reliable information resources; and (vii) information on the duration of special measures (13).

This prospective study aimed to investigate the longitudinal effects of corporate/organizational workplace measures against COVID-19, reported by employees, on reducing psychological distress during a 12-month follow-up between March 2020 and March 2021, during repeated outbreaks of COVID-19 pandemic in Japan. We use data from five follow-up surveys conducted during the follow-up period to know if a changing effect of workplace measures against COVID-19 could remain over the course of the pandemic. Since the first domestic case of COVID-19 was confirmed in January 2020 in Japan, there were three large outbreaks of COVID-19 during the one-year study period – in March and August of 2020 and January in 2021 – for which the government declared the state of emergency, without strict lockdown measures to curb the spread of the disease. The government published guidelines to encourage companies and organizations to implement workplace measures against COVID-19. It was up to each company or organization whether they would take these up or not.

## Methods

### Study design and participants

This study used data from an online longitudinal panel survey of full-time employees in Japan, the Employee Cohort Study in the COVID-19 pandemic in Japan (E-COCO-J) conducted between March 2020 and March 2021. In March 2020, a total of 1448 full-time employees across the whole of Japan were recruited from a large internet panel (>300 000) that included people who were not full-time employees, and they participated in the 1^st^ survey. Following the 1^st^ survey, the 2^nd^ to 6^th^ consecutive follow-up surveys were conducted, inviting the respondents of 1^st^ survey, in May, August, November 2020, and February and March 2021, respectively. The 1^st^ survey was conducted before the 1^st^ outbreak of COVID-19 in April-May 2020 (with the 1^st^ emergency call), and the 2^nd^ survey was immediately after that; the 3^rd^ survey was conducted in the mid of the 2^nd^ outbreak in August 2020, and the 4^th^ survey was conducted between the 2^nd^ and 3^rd^ outbreaks; the 5^th^ and 6^th^ surveys were conducted after a larger outbreak from December 2020 to February 2021 (with the 2^nd^ emergency call in January- March 2021). We limited the subjects to those currently employed, excluding those who were unemployed, temporarily laid-off, on maternity-, childcare- or nursing care-leave, or on long-term sick leave, and those with missing responses to relevant variables at baseline.

The Research Ethics Committee of the Graduate School of Medicine/Faculty of Medicine, University of Tokyo approved this study protocol [No. 10856- (2) (3) (4) (5)]. The study conformed to the Strengthening the Reporting of Observational Studies in Epidemiology (STROBE) guidelines (15, 16).

### Measures

*Workplace measures against COVID-19*. The degree of implementation of workplace measures for prevention and control of COVID-19 was assessed by using a 23-item original scale, which was developed based on workplace measures in the past outbreak of novel influenza and a discussion by occupational health professionals (17–19). Briefly, the scale consisted of the following seven specific categories: (i) facilitating employees’ preventive measures (wearing masks, etc.) (5 items); (ii) taking measures to reduce the risk of infection in the workplace (disinfection of the work environment, etc.) (8 items); (iii) establishing procedures for staying at home and clinical contact (request to refrain from going to work when ill, etc.) (4 items); (iv) establishing rules for temporary leave when infected (providing information on how to deal with infected cases in the workplace, etc.) (3 items); (v) providing accommodation for high-risk people (eg, with chronic conditions) (1 item); (vi) suggesting the access to reliable information sources (1 item); and (vii) informing the duration of these specific measures (1 item). Responses to each item were dichotomized into “implemented” or “not implemented.” We calculated the number of implemented measures in total and by the categories as total and area-specific indicators of implementation of the workplace measures.

We used data from the 2^nd^ wave survey (May 2020) to assess workplace measures against COVID-19 implemented by companies/organizations of participants. This was because the workplace measures substantially changed between the 1^st^ and 2^nd^ wave surveys (19) and using the data of the 2^nd^ wave survey would provide the more stable measurement of the workplace measures. The indicators were stable, with high concordance between the 2^nd^ and 3^rd^ surveys: ICC, 0.769 for total; 0.575, 0.760, 0.637, 0.563, 0.412, 0.488, and 0.382 for the subscales (i–vii). The total and subscale scores were used as continuous variables in the analyses.

*Psychological distress*. Psychological distress in the last 30 days was measured by a corresponding 18-item scale of the Brief Job Stress Questionnaire (BJSQ) (20), which asks about a wide range of psychiatric symptoms, such as lack of vigor, anger-irritability, fatigue, anxiety, and depression. Each item was rated on a four-point Likert scale from 1 (“never”) to 4 (“almost always”). The sum of the item scores was calculated to be a total score (ranging 18–72), with a higher score indicating greater psychological distress (21). High levels of psychological distress are indicative of impaired mental health and may reflect common mental disorders (22). The reliability and validity of the Japanese version of BJSQ have been verified (20). The internal consistency reliability (Cronbach’s alpha coefficient) at the 1^st^ survey was 0.90 in this study. The scale was applied in the 1^st^ to 6^th^ surveys.

### Covariates

The demographic covariates included sex (male or female), age (in years), marital status (single or married), and living in areas under the state of emergency in May 2020 (yes or no). Job-related covariates included company size (≥500 employees, 50–499 employees, and <50 employees), occupation (managers, non-manual workers, manual workers, or healthcare workers), remote work (no or any type of remote work). Health-related covariates included chronic physical conditions (any of ten predetermined physical conditions). Occupation and remote work at the 2^nd^ survey were used because these covariates were not measured at the 1^st^ survey. Otherwise, the covariates were measured at the 1^st^ survey.

### Statistical analysis

The numbers and proportions of participants were tabulated by groups classified based on the baseline covariates. The averages and standard deviations (SD) of the numbers of total and subcategorical workplace measures were calculated. Pearson’s correlation coefficients were calculated between total and subcategorical workplace measures on the one hand, and psychological distress scores at baseline and at each follow-up survey. Multiple linear regression model analysis was conducted of the psychological distress score at each of the 2^nd^ to 6^th^ surveys on scores of the seven subcategorical workplace measures against COVID-19, adjusting for psychological distress score at the 1^st^ survey and the other covariates (sex, age, marital status, company size, occupation, remote work, living in areas under the state of emergency, chronic condition). In this series of analyses, the number of participants may vary depending on their participation in each survey. Finally, a mixed model analysis with repeated measured was conducted of psychological distress scores at the six surveys in the wave of surveys (1^st^–6^th^) with the 1^st^ survey as the reference, and each of scores of the seven subcategorical workplace measures, and their interactions, adjusting for the other covariates (the analysis of variance model). The interaction term between the wave of surveys (scored 1–6) as a continuous variable and a score of subcategorical workplace measures was also estimated to know the linear time trend of the association between workplace measures and psychological distress (the growth model). A mixed model analyses with repeated measured of psychological distress over seven months were conducted in each of the subgroups classified by occupation (managers, non-manual workers, manual workers, or healthcare workers). In addition, similar mixed model analyses with repeated measures of psychological distress over seven months were conducted on the individual scores of the 23 items of workplace measures. Statistical significance was set as a two-sided P<0.05. However, we applied Bonferroni’s method to the five multiple linear regression models to set P<0.01 (=0.05/5) for the statistical significance considering multiple significance tests. All statistical analysis was performed with SPSS 28.0 (IBM Corp, Armonk, NY, USA), the Japanese version.

## Results

### Participants flow

**Figure 1:**
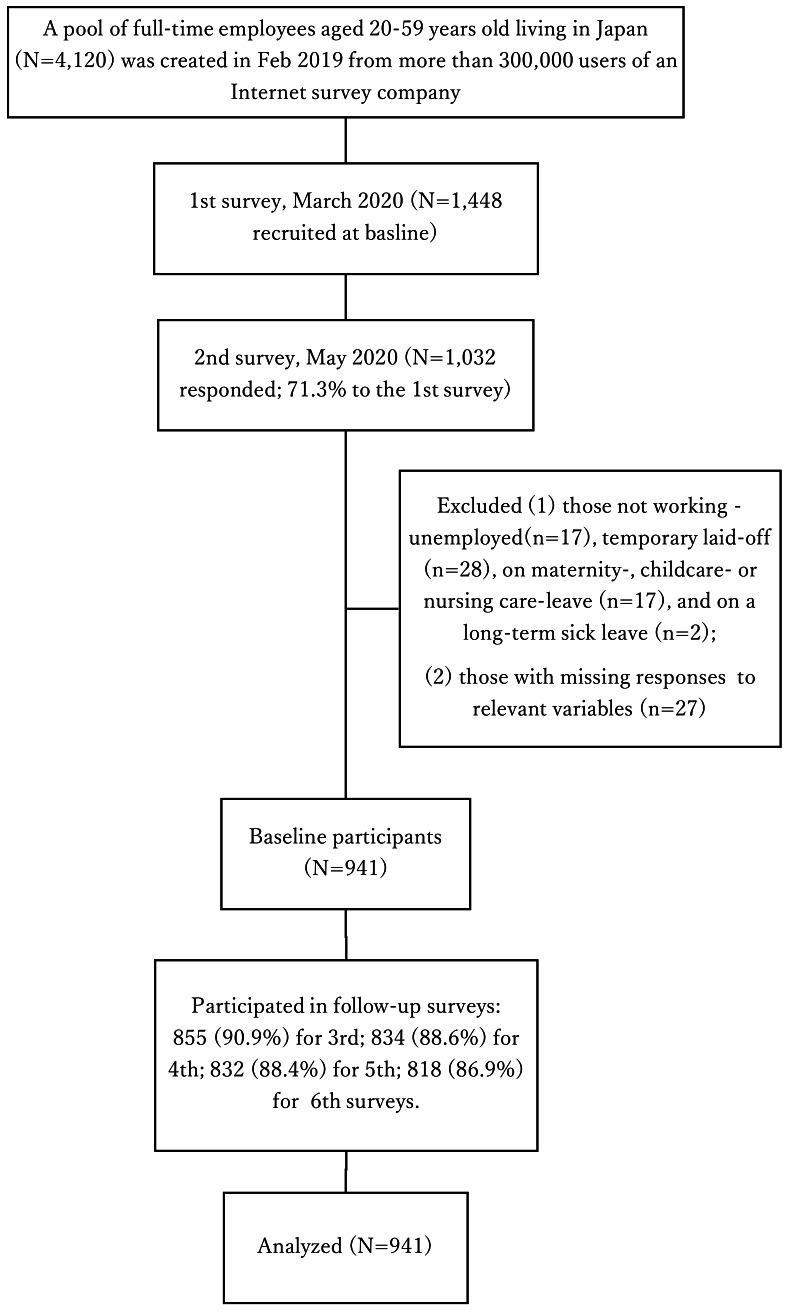
Participant flow chart of a 12-month prospective study in the COVID-19 pandemic.

A total of 1032 responded to the 2^nd^ survey of May 2020 (figure 1). Those who were not working were excluded: unemployed (N=17), temporarily laid-off (N=28), on maternity-, childcare- or nursing care-leave (N=17), and on a long-term sick leave (N=2). In addition, respondents who had missing responses to relevant variables were excluded (N=27). The remaining 941 respondents were used as baseline participants. They participated in the follow-up surveys: 855 (90.9%) for 3^rd^; 834 (88.6%) for 4^th^; 832 (88.4%) for 5^th^; and 818 (86.9%) for 6^th^ surveys.

### Participant baseline characteristics

An equal numbers of participants were distributed in terms of sex and marital status (table 1). The average age was 41.29 years old. About half were on the non-manual job; about 10% were healthcare workers. One-third of the participants engaged in remote work. Most lived in areas with a state of emergency for the COVID-19 pandemic. One in seven had a chronic condition. The average number of the workplace measures against COVID-19 was 14.73.

**Table 1:** Baseline characteristics of the respondents (N=941). [SD=standard deviation; BJSQ=Brief Job Stress Questionnaire]

Variables ^a^	N	%	Average	SD
State of emergency area in May 2020			
No	288	30.6		
Yes	653	69.4		
Sex				
Male	490	52.1		
Female	451	47.9		
Marital status				
Single	462	49.1		
Married	479	50.9		
Age (years)			41.29	10.56
Company size (number of employees)				
≥500	401	42.6		
50–499	341	36.2		
<50	199	21.2		
Occupation ^a^				
Manager	97	10.3		
Non-manual	502	53.3		
Manual	234	24.9		
Healthcare	108	11.5		
Remote work ^a^				
No	654	69.5		
Yes	287	30.5		
Chronic condition				
No	809	86.0		
Yes	132	14.0		
Psychological distress (BJSQ score)			41.18	11.57
Workplace measures against COVID-19 (number of items) ^a^				
Total (23)			14.73	5.77
Encouraging individual-based preventive measures (5)			4.32	1.16
Measures to reduce the risk of infection in the workplace (8)			4.11	2.27
Procedure for staying at home and clinical contact (4)			2.81	1.28
Temporary leave when infected (3)			1.81	1.24
Accommodation of high-risk people (1)			0.59	0.49
Reliable information resources (1)			0.54	0.50
Duration of special measures (1)			0.55	0.50

a Occupation, remote work, and workplace measures against COVID-19 were measured at T2 (May 2020). Otherwise, variables were measured at T1 (March 2020).

### Psychological distress mean scores (baseline to 6^th^ survey)

The mean score for psychological distress was 41.18 (SD 11.57, N=941) in the 1^st^ survey, 41.19 (SD 11.08, N=941) in the 2^nd^ survey, 42.13 (SD 11.65, N=855) in the 3^rd^ survey, 41.02 (SD 11.38, N=834) in the 4^th^ survey, 40.94 (SD 11.47, N=832) in the 5^th^ survey, and 40.83 (SD 11.21, N=818) in the 6^th^ survey.

### Correlations between workplace measures and psychological distress

The total number of the workplace measures against COVID-19 statistically significantly correlated with psychological distress at any of the 1^st^ survey and follow-up surveys (P<0.05) (table 2). Some subcategorical workplace measures showed statistically significantly negative association with psychological distress at some of follow-up surveys but others did not. Facilitating employees’ preventive measures statistically significantly and negatively correlated with psychological distress at the 4^th^ and 5^th^ surveys. Measures to reduce the risk of infection in the workplace statistically significantly and negatively correlated with psychological distress at the 1^st^, 2^nd^, 3^rd^, 5^th^ and 6^th^ surveys. Establishing procedures for staying at home and clinical contact statistically significantly and negatively correlated with psychological distress at all surveys. Establishing rules for temporary leave when infected statistically significantly and negatively correlated with psychological distress at the 3^rd^ survey. None of the following were statistically significantly correlated with psychological distress in any of surveys: providing accommodation for high-risk people, suggesting the access to reliable information sources, or informing the duration of these specific measures.

**Table 2:** Association of workplace measures against COVID-19 ^a^ with psychological distress ^b^ at each of 1^st^ to 6^th^ surveys: Pearson’s correlation coefficients (rs).

Workplace measures against COVID-19 (score)	1^st^ (March 2020) (N=941)		2^nd^ (May 2020) (N=941)		3^rd^ (August 2020) (N=855)		4^th^ (November 2020) (N=834)		5^th^ (February 2021) (N=832)		6^th^ (March 2021)(N=818)	
					
	r	P-value	r	P-value	r	P-value	r	P-value	r	P-value	r	P-value
Total	-0.070	0.031*	-0.066	0.044*	-0.099	0.004**	-0.077	0.025*	-0.101	0.004**	-0.092	0.009**
Encouraging individual-based preventive measures	-0.027	0.403	-0.034	0.298	-0.063	0.065	-0.073	0.034*	-0.081	0.020*	-0.065	0.063
Measures to reduce the risk of infection in the workplace	-0.071	0.030*	-0.071	0.030*	-0.094	0.006**	-0.065	0.059	-0.102	0.003**	-0.097	0.006**
Procedure for staying at home and clinical contact	-0.092	0.005**	-0.074	0.023*	-0.122	<0.001**	-0.090	0.009**	-0.106	0.002**	-0.111	0.001**
Temporary leave when infected	-0.044	0.178	-0.043	0.183	-0.071	0.039*	-0.049	0.155	-0.053	0.128	-0.045	0.202
Accommodation of high-risk people	-0.062	0.059	-0.032	0.321	-0.036	0.298	-0.019	0.574	-0.040	0.251	-0.042	0.232
Reliable information resources	-0.027	0.404	-0.025	0.452	-0.011	0.753	-0.019	0.590	-0.031	0.367	-0.022	0.524
Duration of special measures	0.003	0.917	-0.003	0.923	-0.033	0.342	-0.031	0.377	-0.041	0.236	-0.009	0.787

* P<0.05.

** P<0.01.

a Workplace measures against COVID-19 were measured at T2 (May 2020).

b Psychological distress was measured by a corresponding 18-item scale of the Brief Job Stress Questionnaire (BJSQ).

### Longitudinal association between workplace measures and psychological distress

Multiple linear regression models revealed that facilitating employees’ preventive measures statistically significantly and negatively correlated with psychological distress at the 5^th^ survey (b=-0.819, SE 0.311, P=0.009; after Bonferroni’s correction) (table 3). No other subcategorical workplace measure against COVID-19 statistically significantly correlated with psychological distress from the 2^nd^ to 6^th^ surveys. Psychological distress (1^st^) was correlated with psychological distress from the 2^nd^ to 6^th^ follow-up surveys (P<0.001; after Bonferroni’s correction)

**Table 3a:** Association of workplace measures against COVID-19 ^a^ with psychological distress ^b^ at the 2^nd^ to 4^th^ surveys: multiple linear regression.^c^ [SE=standard error].

	2^nd^ (May 2020) (N=941)				3^rd^ (August 2020) (N=855)				4^th^ (November 2020) (N=834)			
		
	b	SE	beta	P-value	b	SE	beta	P-value	b	SE	beta	P-value
Psychological distress (1^st)^	0.637	0.024	0.665	<0.001*	0.733	0.024	0.728	<0.001*	0.701	0.025	0.704	<0.001*
State of emergency area in May 2020 (yes)	0.776	0.609	0.032	0.203	0.354	0.620	0.014	0.568	1.027	0.631	0.042	0.104
Sex (female)	0.249	0.615	0.011	0.685	-0.658	0.625	-0.028	0.293	0.336	0.635	0.015	0.597
Marital status (married)	0.465	0.605	0.021	0.443	-0.546	0.615	-0.023	0.374	0.238	0.629	0.010	0.706
Age (years)	-0.026	0.028	-0.024	0.365	-0.004	0.029	-0.004	0.883	-0.036	0.029	-0.033	0.706
Company size (≥500 employees as a reference)											
50–499	0.140	0.829	0.006	0.866	-0.146	0.840	-0.006	0.862	0.359	0.852	0.016	0.674
<50	0.325	0.783	0.014	0.678	0.575	0.797	0.024	0.471	0.353	0.805	0.015	0.661
Occupation (manager as a reference)											
Non-manual	0.978	1.000	0.044	0.978	0.548	0.991	0.023	0.581	1.697	1.002	0.074	0.091
Manual	0.546	1.084	0.021	0.504	-0.529	1.083	-0.020	0.625	1.666	1.091	0.063	0.127
Health care	3.048	1.262	0.088	0.016	2.309	1.265	0.063	0.068	4.855	1.299	0.129	<0.001*
Remote work (yes)	-0.971	0.684	-0.040	0.156	-1.124	0.696	-0.044	0.106	-1.302	0.705	-0.053	0.065
Chronic condition (yes)	1.388	0.808	0.044	0.086	0.750	0.810	0.022	0.354	0.494	0.825	0.015	0.549
Workplace measures against COVID-19 (score)											
Encouraging individual-based preventive measures	-0.125	0.290	-0.013	0.666	-0.274	0.294	-0.027	0.352	-0.706	0.294	-0.073	0.016
Measures to reduce the risk of infection in the workplace	-0.089	0.173	-0.018	0.609	-0.032	0.175	-0.006	0.854	0.079	0.177	0.016	0.657
Procedure for staying at home and clinical contact	0.030	0.336	0.003	0.929	-0.500	0.338	-0.054	0.140	-0.076	0.342	-0.009	0.823
Temporary leave when infected	-0.207	0.320	-0.023	0.518	-0.274	0.318	-0.029	0.388	-0.067	0.320	-0.007	0.834
Accommodation of high-risk people	0.762	0.710	0.034	0.284	1.396	0.709	0.059	0.049	1.526	0.724	0.066	0.035
Reliable information resources	-0.180	0.725	-0.008	0.803	1.095	0.729	0.047	0.134	0.531	0.737	0.023	0.471
Duration of special measures	0.350	0.738	0.016	0.636	-0.681	0.732	-0.029	0.352	-0.822	0.743	-0.036	0.269

* Significant after the Bonferroni’s correction, P<0.01 (=0.05/5 waves). See the text for the detail.

a Workplace measures against COVID-19 were measured at T2 (May 2020).

b Psychological distress was measured by a corresponding 18-item scale of the Brief Job Stress Questionnaire (BJSQ).

c Adjusting for psychological distress and other covariates. Occupation, remote work, and workplace measures against COVID-19 were measured at T2 (May 2020). Otherwise, variables were measured at T1 (March 2020).

**Table 3b:** Association of workplace measures against COVID-19 ^a^ with psychological distress ^b^ at the 5^th^ to 6^th^ surveys: multiple linear regression ^c^.

	5^th^ (February 2021) (N=832)				6^th^ (March 2021) (N=818)			
	
	b	SE	beta	P-value	b	SE	beta	P-value
Psychological distress (1^st^)	0.684	0.026	0.681	<0.001*	0.662	0.025	0.676	<0.001*
State of emergency area in May 2020 (yes)	0.253	0.653	0.010	0.698	-0.387	0.638	-0.016	0.545
Sex (female)	0.842	0.664	0.037	0.205	0.279	0.649	0.012	0.668
Marital status (married)	-0.559	0.652	-0.024	0.392	-0.999	0.635	-0.045	0.116
Age (years)	-0.010	0.030	-0.009	0.315	-0.021	0.030	-0.019	0.488
Company size (≥500 employees as a reference)								
50–499	-0.401	0.891	-0.017	0.653	1.148	0.866	0.051	0.185
<50	0.888	0.840	0.037	0.290	2.052	0.816	0.088	0.012
Occupation (manager as a reference)							
Non-manual	1.223	1.032	0.053	0.236	1.939	1.008	0.086	0.055
Manual	2.156	1.135	0.080	0.058	1.831	1.104	0.070	0.098
Health care	4.238	1.331	0.114	0.002*	5.124	1.298	0.140	<0.001*
Remote work (yes)	-0.151	0.732	-0.006	0.836	-1.069	0.708	-0.044	0.131
Chronic condition (yes)	0.875	0.857	0.027	0.307	1.999	0.836	0.062	0.017
Workplace measures against COVID-19 (score)								
Encouraging individual-based preventive measures	-0.819	0.311	-0.082	0.009*	-0.508	0.298	-0.053	0.089
Measures to reduce the risk of infection in the workplace	-0.110	0.185	-0.022	0.552	-0.104	0.180	-0.021	0.565
Procedure for staying at home and clinical contact	0.148	0.358	0.016	0.679	-0.465	0.348	-0.053	0.182
Temporary leave when infected	0.322	0.340	0.035	0.343	0.334	0.329	0.037	0.311
Accommodation of high-risk people	0.821	0.754	0.035	0.276	0.625	0.735	0.028	0.395
Reliable information resources	0.176	0.783	0.008	0.822	0.191	0.754	0.009	0.800
Duration of special measures	-0.938	0.778	-0.041	0.228	0.033	0.754	0.001	0.965

* Significant after the Bonferroni’s correction, P<0.01 (=0.05/5 waves). See the text for the detail.

a Workplace measures against COVID-19 were measured at T2 (May 2020).

b Psychological distress was measured by a corresponding 18-item scale of the Brief Job Stress Questionnaire (BJSQ).

c Adjusting for psychological distress and other covariates. Occupation, remote work, and workplace measures against COVID-19 were measured at T2 (May 2020). Otherwise, variables were measured at T1 (March 2020).

### Mixed model analysis of changing associations between workplace measures and psychological distress over time

Table 4 shows the results of mixed model analysis of associations between workplace measures against COVID-19 and psychological distress over time, testing interaction terms between each score of subcategorical workplace measures and survey waves. The results showed that facilitating employees’ preventive measures were statistically significantly and negatively associated with psychological distress at the 5^th^ compared to 1^st^ survey as a reference (b=-0.518, SE=0.259, P=0.046) (table 4). The coefficients for these interaction terms tended to be greater for the later surveys. The interaction between the number of the subcategorical workplace measures facilitating employees’ preventive measures and the order of wave (as a continuous variable) was statistically significant and negative (b=-0.096, SE 0.047, P=0.041). No other subcategorical workplace measure showed statistically significant interactions with waves (compared to the 1^st^ survey) for psychological distress.

**Table 4a:** Changing associations between workplace measures ^a^ and psychological distress ^b^ over time: mixed model analysis. ^c^ [SE=standard error].

ANOVA model	Total score			Facilitating employees’ preventive practices			Taking measures to reduce the risk of infection in the workplace			Establishing procedures for staying at home and clinical contact		
			
	b	SE	P-value	b	SE	P-value	b	SE	P-value	b	SE	P-value
Main effect of wave	-0.084	0.072	0.239	-0.130	0.329	0.693	-0.170	0.183	0.351	-0.648	0.311	0.037*
Interaction with wave												
1^st^ (March 2020, reference)	.	.	.	.	.	.	.	.	.	.	.	.
2^nd^ (May 2020)	0.015	0.046	0.742	-0.052	0.229	0.821	0.014	0.117	0.907	0.192	0.208	0.357
3^rd^ (August 2020)	-0.059	0.051	0.250	-0.359	0.254	0.157	-0.111	0.129	0.388	-0.272	0.230	0.239
4^th^ (November 2020)	0.001	0.052	0.981	-0.395	0.256	0.123	0.040	0.131	0.761	0.104	0.234	0.658
5^th^ (February 2021)	-0.060	0.052	0.250	-0.518	0.259	0.046*	-0.189	0.132	0.153	-0.037	0.234	0.873
6^th^ (March 2021)	-0.035	0.052	0.498	-0.398	0.259	0.124	-0.124	0.132	0.350	-0.152	0.235	0.519
Growth model												
Main effect	-0.073	0.072	0.308	-0.070	0.330	0.833	-0.128	0.184	0.484	-0.568	0.313	0.069
Interaction with wave	-0.009	0.009	0.321	-0.096	0.047	0.041*	-0.029	0.024	0.219	-0.030	0.043	0.476

* P<0.05.

**P<0.01.

a Workplace measures against COVID-19 were measured at T2 (May 2020).

b Psychological distress was measured by a corresponding 18-item scale of the Brief Job Stress Questionnaire (BJSQ).

c Adjusting for psychological distress and other covariates. Occupation, remote work, and workplace measures against COVID-19 were measured at T2 (May 2020). Otherwise, variables were measured at T1 (March 2020).

**Table 4b:** Changing associations between workplace measures ^a^ and psychological distress ^b^ over time: mixed model analysis ^c^.

ANOVA model	Establishing rules for temporary leave when infected			Providing accommodation for high-risk people			Suggesting the access to reliable information sources			Informing the duration of these specific measures		
			
	b	SE	P-value	b	SE	P-value	b	SE	P-value	b	SE	P-value
Main effect of wave	-0.230	0.310	0.458	-0.740	0.780	0.343	-0.198	0.763	0.796	0.716	0.773	0.354
Interaction with wave												
1^st^ (March 2020, reference)	.	.	.	.	.	.	.	.	.	.	.	.
2^nd^ (May 2020)	0.021	0.214	0.920	0.719	0.540	0.183	0.087	0.533	0.871	-0.150	0.534	0.780
3^rd^ (August 2020)	-0.258	0.236	0.275	0.526	0.593	0.375	0.227	0.587	0.699	-0.923	0.588	0.117
4^th^ (November 2020)	0.022	0.240	0.927	1.049	0.603	0.082	0.314	0.597	0.599	-0.682	0.598	0.254
5^th^ (February 2021)	-0.016	0.241	0.948	0.356	0.606	0.556	-0.215	0.599	0.719	-1.071	0.600	0.074
6^th^ (March 2021)	0.133	0.242	0.583	0.357	0.609	0.558	0.209	0.603	0.729	-0.385	0.604	0.524
Growth model												
Main effect	-0.319	0.311	0.306	-0.423	0.784	0.589	-0.146	0.767	0.849	0.639	0.777	0.411
Interaction with wave	0.023	0.044	0.603	0.048	0.110	0.664	0.014	0.109	0.899	-0.124	0.109	0.257

a Workplace measures against COVID-19 were measured at T2 (May 2020).

b Psychological distress was measured by a corresponding 18-item scale of the Brief Job Stress Questionnaire (BJSQ).

c Adjusting for psychological distress and other covariates. Occupation, remote work, and workplace measures against COVID-19 were measured at T2 (May 2020). Otherwise, variables were measured at T1 (March 2020).

In the analyses among subgroups by occupation, facilitating employees’ preventive measures (5^th^ survey in ANOVA model b=-2.374, SE 1.103, P=0.032) and informing the duration of these specific measures (3^th^ survey in ANOVA model b=-4.394, SE 2.002, P=0.029) were significantly and negatively associated with psychological distress, compared to the 1^st^ survey as a reference, among healthcare workers. Taking measures to reduce the risk of infection in the workplace 4^th^ survey in ANOVA model: b=0.597, SE 0.265, P=0.025) were significantly associated with psychological distress, compared to the 1^st^ survey as a reference, among manual workers. None of the interactions between the number of the workplace measures and the order of wave (as a continuous variable) were statistically significant in any occupational group (data available on request).

Similar mixed model analyses using each of the 23 items of workplace measures against COVID-19 showed that encourage hand alcohol disinfection (5^th^ survey in ANOVA model: b=-2.579, SE 1.150, P=0.025, 6^th^ survey in ANOVA model: b=-2.508, SE 1.142, P=0.028, and interaction with wave in the growth model: b=-0.528, SE 0.207, p=0.011) and encourage mask wearing (4^th^ survey in ANOVA model: b=-2.859, SE 1.315, P=0.030) – categorized as facilitating employees’ preventive measures – were significantly and negatively associated with psychological distress (data available on request).

## Discussion

This prospective study found that a subcategorical workplace measure against COVID-19, ie, facilitating employees’ preventive practices, was negatively associated with psychological distress at the follow-up surveys among full-time employees in Japan. The degree of the association tended to be larger at later follow-ups, which was tested to be statistically significant by using a mixed model analysis. The study provides the first prospective evidence for the protective effect of a workplace measure against COVID-19 on the psychological distress of employees.

The score of the subscale (a) facilitating employees’ preventive measures at the 2^nd^ survey (May 2020) was significantly and negatively associated with psychological distress at several follow-ups. The finding is consistent with the observation by Tan et al (12) that workers were less stressed if doing preventive practices, such as hand washing and returning to a prevention-conscious workplace (12). The finding is also in part consistent with our previous cross-sectional finding of the negative association between the number of workplace measures against COVID-19 and psychological distress in a sample of full-time employees, who were the sample of the first survey of this study (13). A greater number of control actions against COVID-19 has been associated with a greater sense of being protected against COVID-19 of employees (23). Such sense may reduce the psychological threat of COVID-19 and thus improve the psychological distress of employees. An established uniform policy of companies/organizations to facilitate all employees doing hygiene practices against COVID-19 in the workplace may be beneficial for employees sharing the same view on how to protect against COVID-19, otherwise the views vary among individual employees which may create tension and discordance between them. Mutual understanding and trust in the workplace have been associated with reduced psychological distress for employees before the COVID-19 pandemic (24). Lastly, individuals often reported that they used hygiene practices to prevent COVID-19 as a coping strategy for psychological stress due to fear of COVID-19 (25). Psychosocial mechanisms underlying the protective effect of the workplace measures against COVID-19 to facilitate employee hygiene practices against COVID-19 should be investigated further. Hygiene practices facilitated by companies/organizations may reduce the psychological distress of employees. However, of course, such a uniform policy should be carefully designed and implemented because it may increase the wrong feelings of being safe among employees while they do not comply with these measures against COVID-19, and peer pressure to comply with these measures may lead to COVID-19-related bullying and harassment.

It is interesting that the negative associations (coefficients) between the workplace measures against COVID-19 to facilitate employee’s hygiene practices and psychological distress were greater for the later follow-ups, such as the 5^th^ survey. The mixed model analysis showed a statistically significant negative interaction between the workplace measures against COVID-19 to facilitate employee’s hygiene practices and the survey waves on psychological distress, which means that the effect of this type of workplace measures against COVID-19 on psychological distress increased along with the time since the baseline. During the follow-up period, the 3^rd^ survey (August 2020) was conducted in the mid of the second outbreak of COVID-19, and the 5^th^ and 6^th^ surveys (February and March 2021) were conducted soon after the third, severer outbreak in Japan. The observed finding that the association between the workplace measure against COVID-19 to facilitate hygiene practices and psychological distress was larger for the later (5^th^ and 6^th^) surveys may imply that the workplace measures could have a larger impact on psychological distress in a severer outbreak where workers may feel a high threat to the infection. As the COVID-19 pandemic becomes severe and prolonged, the workplace measures may be more important for preventing and reducing the psychological distress of employees.

Among individual items of the subscale facilitating employees’ preventive measures, encouraging hand alcohol disinfection showed the most consistent patterns of negative associations with psychological distress at follow-ups. Wearing masks showed such a pattern only at the 4^th^ follow-up; none of the other individual items of the workplace measures was statistically significantly associated with psychological distress at follow-ups. Encouraging hand alcohol disinfection may be effective as a workplace measure to reduce psychological distress during the COVID-19 outbreaks. However, it should be noted that another item – encouraging hygiene such as hand washing and mouth gargling– was not that effective. Employees may perceive encouraging hand alcohol disinfection as a more effective environmental measure because alcohol-based hand sanitizer was prepared by the company and visible in the workplace. This may create a sense of being protected from infection in the workplace among the employees. On the other hand, the effectiveness of hand washing and gargling depends on the individual’s behaviors, even if they are encouraged in the workplace. Employees often may not know how many of their colleagues follow the measure. Even in the same category of hand hygiene encouragement, an environmental approach such as preparation and use of alcohol hand sanitizer may be more effective than encouraging individual hygiene behaviors in reducing psychological distress during the COVID-19 outbreaks. This should be investigated further to establish specific preventive measures against COVID-19 that also protect employees’ mental health.

The total or other subscale scores of workplace measures against COVID-19 did not significantly correlate with psychological distress at any time point of the follow-up in this sample. The number of workplace measures against COVID-19 may be too simple for these areas. The quality, not the number, of workplace measures against COVID-19 may be more important. Compared to facilitating hygiene practices, some measures may not be relevant to some types of industries or workplace structures. The results of the subgroup analysis by occupation showed no clear pattern of the association between the workplace measures and psychological distress over time. This may be attributable to the small sample size in each occupational group. While an effect of facilitating employees’ preventive measures on psychological distress at 5^th^ wave observed in the total sample was replicated among healthcare workers, different types of workplace measures were associated with reduced psychological distress, ie, taking measures to reduce the risk of infection in the workplace among manual works and informing the duration of these specific measures among healthcare workers. The effect of workplace measures against COVID-19 on improving psychological distress may vary among the type of occupations. This should be further investigated with a larger sample of each occupation. Further research is also required using a scale of quantity and quality of workplace measures against COVID-19 and focusing on differences by types of occupation/industry or workplace structures. Moreover, we associated the workplace measures against COVID-19 at the 2^nd^ wave survey (May 2020) with psychological distress at follow-ups, while the implementation of the workplace measures fluctuated, depending on the severity of outbreaks and eflecting the government and company policies (26). This may attenuate the observed association between the workplace measures and psychological distress over time. It would be better if the workplace measures at every time point or their changes over time with psychological distress at follow-ups. Such a statistical analysis is quite complex and possibly beyond the scope of this study. Future studies could also employ a more sophisticated statistical modeling to investigate the association between the workplace measures and psychological distress both at multiple points of time.

### Limitations

The study sample may not be representative of the working population in Japan. Our sample consisted only of full-time employees recruited from an internet survey company. Thus, they were more likely to be managers and non-manual workers compared to the national labor statistics in Japan (27, 28). The scale of workplace measures against COVID-19 was developed to be Japan-specific. Thus, it may not be applicable to other countries and the generalizing of findings in this study may be limited.

While the follow-up rate was relatively high (>84%), attrition may result in selection bias. For instance, if participants with high psychological distress who were dissatisfied with corporate/organizational workplace COVID-19 measures were more likely to drop out of the study, the association between workplace measures and psychological distress may be overestimated. Participants who were frustrated with their general working conditions may under-report the workplace measures against COVID-19 and over-report psychological distress. Other factors associated with both negative reporting of workplace measures and psychological distress, such as neuroticism personality, may confound the findings, although we adjusted for psychological distress at baseline to minimize such bias. In addition, psychological distress at 1^st^ survey was a strong predictor of psychological distress at follow-ups. Adjusting psychological at 1^st^ survey may lead to the limited statistical power to identify the effect of the workplace measures on psychological distress at follow-ups.

The scale of workplace measures was self-report and thus may be unreliable because employees may not be fully informed of the measures or able to recall exactly the measures taken. If employees are unaware of the implementation of the workplace measures against COVID-19, they may report a lower number of workplace measures being implemented in their own company, or the COVID-19 measures may be affecting their mental health. In addition, the scale of workplace measures against COVID-19 was developed as a formative scale (29), based on previous studies and through discussion among professions. Only face validity was tested and other psychometric properties were not tested (29). Some subcategory scales included only one item. The scale may be limited in its predictive ability.

### Concluding remarks

This prospective study found that an area of workplace measures against COVID-19, ie, facilitating employees’ preventive practices, was negatively associated with psychological distress at some of the time points of the 12-month follow-up among full-time employees in Japan. Among individual items of the workplace measures, this negative association was more clearly observed for encouraging hand alcohol disinfection and wearing masks – both of which are categorized as facilitating employees’ preventive practices. The association became stronger at later follow-ups. The study provides the first prospective evidence for the protective effect of workplace measures against COVID-19 on the psychological distress of employees.

## Supplementary material

Supplementary material

## References

[1] Holmes EA, O'Connor RC, Perry VH, Tracey I, Wessely S, Arseneault L (2020). Multidisciplinary research priorities for the COVID-19 pandemic:a call for action for mental health science. Lancet Psychiatry.

[2] Rajkumar RP (2020). COVID-19 and mental health:A review of the existing literature. Asian J Psychiatr.

[3] Vigo D, Patten S, Pajer K, Krausz M, Taylor S, Rush B (2020). Mental Health of Communities during the COVID-19 Pandemic. Can J Psychiatry.

[4] Cénat JM, Blais-Rochette C, Kokou-Kpolou CK, Noorishad PG, Mukunzi JN, McIntee SE (2021). Prevalence of symptoms of depression, anxiety, insomnia, posttraumatic stress disorder, and psychological distress among populations affected by the COVID-19 pandemic:A systematic review and meta-analysis. Psychiatry Res.

[5] Sasaki N, Kuroda R, Tsuno K, Kawakami N (2020). The deterioration of mental health among healthcare workers during the COVID-19 outbreak:A population-based cohort study of workers in Japan. Scand J Work Environ Health.

[6] Xiong J, Lipsitz O, Nasri F, Lui LM, Gill H, Phan L (2020). Impact of COVID-19 pandemic on mental health in the general population:A systematic review. J Affect Disord.

[7] Giorgi G, Lecca LI, Alessio F, Finstad GL, Bondanini G, Lulli LG (2020). COVID-19-Related Mental Health Effects in the Workplace:A Narrative Review. Int J Environ Res Public Health.

[8] International Labour Organization (2020). Managing work-related psychosocial risks during the COVID-19 pandemic.

[9] Flaxman S, Mishra S, Gandy A, Unwin HJ, Mellan TA, Coupland H (2020). Imperial College COVID-19 Response Team. Estimating the effects of non-pharmaceutical interventions on COVID-19 in Europe. Nature.

[10] CDC (2020). Guidance for businesses and employers responding to coronavirus disease 2019 (COVID-19).

[11] Miyaki K, Sakurazawa H, Mikurube H, Nishizaka M, Ando H, Song Y (2011). An effective quarantine measure reduced the total incidence of influenza A H1N1 in the workplace:another way to control the H1N1 flu pandemic. J Occup Health.

[12] Tan W, Hao F, McIntyre RS, Jiang L, Jiang X, Zhang L (2020). Is returning to work during the COVID-19 pandemic stressful?A study on immediate mental health status and psychoneuroimmunity prevention measures of Chinese workforce. Brain Behav Immun.

[13] Sasaki N, Kuroda R, Tsuno K, Kawakami N (2020). Workplace responses to COVID-19 associated with mental health and work performance of employees in Japan. J Occup Health.

[14] Yasuda Y, Ishimaru T, Nagata M, Tateishi S, Eguchi H, Tsuji M (2021). CORoNaWork project. A cross-sectional study of infection control measures against COVID-19 and psychological distress among Japanese workers. J Occup Health.

[15] Vandenbroucke JP, von Elm E, Altman DG, Gøtzsche PC, Mulrow CD, Pocock SJ (2007). STROBE Initiative. Strengthening the Reporting of Observational Studies in Epidemiology (STROBE):explanation and elaboration. PLoS Med.

[16] von Elm E, Altman DG, Egger M, Pocock SJ, Gøtzsche PC, Vandenbroucke JP (2014). STROBE Initiative. The Strengthening the Reporting of Observational Studies in Epidemiology (STROBE) Statement:guidelines for reporting observational studies. Int J Surg.

[17] Sasaki N, Kuroda R, Tsuno K, Kawakami N (2020). Workplace responses to COVID-19 and their association with company size and industry in an early stage of the epidemic in Japan. Environ Occup Health Practice.

[18] Wada K, Suzuki H, Imai T, Aizawa Y (2012). [A tool for supporting decision making for occupational health practitioners at the occurrence of novel influenza]. Sangyo Eiseigaku Zasshi.

[19] Sasaki N, Imamura K, Kataoka M, Kuroda R, Tsuno K, Sawada U (2020). COVID-19 measurements at the workplace in various industries and company sizes:a 2-month follow-up cohort study of full-time employees in Japan. Environ Occup Health Practice.

[20] Shimomitsu T, Haratani T, Nakamura K, Kawakami N, Hayashi T, Hiro H (2000). Final development of the Brief Job Stress Questionnaire mainly used for assessment of the individuals. The Ministry of Labor sponsored grant for the prevention of work-related illness, FY 1999 report.

[21] Wada K, Sairenchi T, Haruyama Y, Taneichi H, Ishikawa Y, Muto T (2013). Relationship between the onset of depression and stress response measured by the Brief Job Stress Questionnaire among Japanese employees:a cohort study. PLoS One.

[22] Sieber SD (1974). Toward a theory of role accumulation. Am Sociol Rev.

[23] Ceryes C, Robinson J, Biehl E, Wirtz AL, Barnett DJ, Neff R (2021). Frequency of Workplace Controls and Associations With Safety Perceptions Among a National Sample of US Food Retail Workers During the COVID-19 Pandemic. J Occup Environ Med.

[24] Eguchi H, Tsutsumi A, Inoue A, Hikichi H, Kawachi I (2018). Association of workplace social capital with psychological distress:results from a longitudinal multilevel analysis of the J-HOPE Study. BMJ Open.

[25] Minisry of Health L, and Welfare (2020). The results of the national mental health survey in the COVID-19 pandemic.

[26] Asaoka H, Sasaki N, Imamura K, Kuroda R, Tsuno K, Kawakami N (2021). Changes in COVID-19 measures in the workplace:8-month follow-up in a cohort study of full-time employees in Japan. J Occup Health.

[27] Ministry of Health L, and Welfare (2021). The results of the Basic Survey on Wage Structure in 2020.

[28] The Japan Institute for Labour Policy and Training (2021). Number of employees by occupation in 2020.

[29] Diamantopoulos A, Siguaw JA (2006). Formative versus reflective indicators in organizational measure development:A comparison and empirical illustration. Br J Manage.

