# Supplementary material for: Effects of workplace measures against COVID-19 on psychological distress of full-time employees: A 12-month prospective study in the COVID-19 pandemic
Source: Scand J Work Environ Health. 2022 Aug 31;48(6):468–78. doi: 10.5271/sjweh.4030 (PMC9888436; doi:10.5271/sjweh.4030)
Supplement: Supplementary material [file SJWEH-48-468-S001.pdf]

Effects of workplace measures against COVID-19 on psychological distress of full-time employees: A 12-month prospective study in the COVID-19 pandemic[1]

by Hiroki Asaoka, MSN, Natsu Sasaki, MD, PhD, Kotaro Imamura, PhD, Reiko Kuroda, MD, PhD, Kanami Tsuno, PhD, Norito Kawakami, MD, PhD [2]

1. Supplementary material
2. Correspondence to: Norito Kawakami, Department of Mental Health, Graduate School of Medicine, The University of Tokyo, 7-3-1, Hongo, Bunkyo-ku, Tokyo 113-0033, Japan. Junpukai Foundation, 2-3-1, Daiku, Kita-ku, Okayama 700-0913, Japan. [E-mail: nkawakami@m.u-tokyo.ac.jp]

Supplement Table 1. Association of total score of workplace measures against COVID-19[a] with psychological distress[b] at the 2nd to 6th surveys: multiple linear regression[c].

	2nd (May 2020) (N=941)				3rd (August 2020) (N=855)				4th (November 2020) (N=834)				5th (February 2021) (N=832)				6th (March 2021) (N=818)			
	b	SE	beta	p	b	SE	beta	p	b	SE	beta	p	b	SE	beta	p	b	SE	beta	p
Psychological distress (1st)	0.627	0.024	0.665	<0.001*	0.734	0.024	0.729	<0.001*	0.698	0.025	0.702	<0.001*	0.679	0.026	0.676	<0.001*	0.662	0.025	0.676	<0.001*
Emergency call area (yes)	0.809	0.604	0.034	0.181	0.475	0.617	0.019	0.441	1.237	0.628	0.050	0.049	0,421	0.650	0.017	0.517	-0.211	0.635	-0.009	0.740
Sex (female)	0.221	0.610	0.010	0.717	-0.873	0.620	-0.037	0.160	0.120	0.632	0.005	0.849	0.699	0.661	0.030	0.291	0.140	0.645	0.006	0.828
Marital status (married)	0.443	0.601	0.020	0.461	-0.615	0.612	-0.026	0.316	0.212	0.628	0.009	0.736	-0.582	0.650	-0.025	0.371	-0.999	0.632	-0.045	0.114
Age (years)	-0.024	0.028	-0.023	0.384	-0.001	0.029	-0.001	0.977	-0.032	0.029	-0.029	0.276	-0.007	0.030	-0.006	0.827	-0.022	0.030	-0.021	0.450
Company size (500 or more employees as a reference)																				
50-499	0.125	0.824	0.006	0.880	-0.255	0.838	-0.011	0.761	0.340	0.852	0.015	0.690	-0.449	0.889	-0.019	0.614	0.958	0.860	0.042	0.266
Less than 50	0.337	0.778	0.015	0.665	0.471	0.793	0.019	0.553	0.333	0.805	0.014	0.679	0.814	0.837	0.034	0.331	1.864	0.811	0.080	0.022
Occupation (manager as a reference)																				
Non-manual	0.976	0.996	0.044	0.328	0.553	0.991	0.024	0.577	1.725	1.003	0.076	0.086	1.287	1.033	0.056	0.213	1.973	1.007	0.088	0.050
Manual	0.577	1.079	0.023	0.593	-0.659	1.081	-0.024	0.543	1.567	1.092	0.060	0.152	2.134	1.132	0.080	0.060	1.782	1.100	0.068	0.106
Health care	3.066	1.256	0.088	0.015	2.425	1.264	0.066	0.055	4.916	1.300	0.131	<0.001*	4.353	1.330	0.117	0.001*	5.212	1.296	0.143	<0.001*
Remote work (yes)	-0.891	0.670	-0.037	0.184	-1.045	0.685	-0.041	0.127	-1.141	0.694	-0.046	0.100	-0.136	0.720	-0.005	0.850	-1.009	0.695	-0.042	0.147
Chronic condition (yes)	1.380	0.804	0.043	0.086	0.800	0.810	0.024	0.323	0.503	0.826	0.015	0.542	0.901	0.857	0.027	0.294	2.045	0.836	0.064	0.015
Workplace measures against COVID-19 (total score)	-0.025	0.054	-0.013	0.646	-0.085	0.055	-0.042	0.726	-0.031	0.056	-0.016	0.583	-0.061	0.058	-0.031	0.294	-0.083	0.056	-0.043	0.140

* Significant after the Bonferroni's correction, p<0.01 (=0.05/5 waves) See the text for the detail.

SE: standard error.

[a] Workplace measures against COVID-19 were measured at T2 (May 2020).

[b] Psychological distress was measured by a corresponding 18-item scale of the Brief Job Stress Questionnaire (BJSQ).

[c] Adjusting for psychological distress and other covariates. Occupation, remote work, and workplace measures against COVID-19 were measured at T2 (May 2020). Otherwise, variables were measured at T1 (March 2020).

Supplement Table 2. The list of the 23 items of workplace measures against COVID-19.

(a) Encouraging individual-based preventive measures
Hand washing, gargle enforcement
Enforce cough etiquette
Encourage hand alcohol disinfection
Encourage wearing masks
Enforcement of temperature measurement
(b) Taking measures to reduce the risk of infection in the workplace
Refrain from traveling overseas
Changing the working environment (desk layout, flow lines, installing vinyl curtains, etc.)
Encourage telework and telecommuting (including remote work)
Enforcement of staggered work
Disinfection of the work environment
Restrictions on the use of employee cafeterias
Restrictions on eating, drinking, and entertainment for work
Cancel or postpone internal or external business events
(c) Establishing for staying at home and clinical contact
Request to refrain from going to work when ill
Waiting at home if you have a history[+] of staying abroad
Report request for fever
Dissemination of information on home remedies and consultations for COVID-19
(d) Establishing rules for temporary leave when infected
Providing information on how to deal with infected cases in the workplace
Providing information on compensation when waiting at home
Provision of information on compensation when taking leave due to infection
(e) Providing accommodation of high-risk people
Consideration for staff who are at high risk of serious illness in case of infection (elderly people, pregnant women, etc.)
(f) Suggesting the access to reliable information resources
Announcement of reliable information collection destinations (such as the Ministry of Health, Labor and Welfare website)
(g) Informing the duration of special measures
Providing information on how long special measures will be taken

[+] The item was not restricted to any countries or any period about the history of staying abroad.
